# A Randomized Trial of Personalized Cognitive-Behavior Therapy for Alcohol Use Disorder in a Public Health Clinic

**DOI:** 10.3389/fpsyt.2018.00297

**Published:** 2018-07-10

**Authors:** Jason M. Coates, Matthew J. Gullo, Gerald F. X. Feeney, Ross M. Young, Jason P. Connor

**Affiliations:** ^1^Centre for Youth Substance Abuse Research, The University of Queensland, Brisbane, QLD, Australia; ^2^School of Psychology, The University of Queensland, Brisbane, QLD, Australia; ^3^Alcohol and Drug Assessment Unit, Division of Medicine, Princess Alexandra Hospital, Brisbane, QLD, Australia; ^4^Faculty of Health, Queensland University of Technology, Brisbane, QLD, Australia; ^5^Faculty of Medicine, The University of Queensland, Brisbane, QLD, Australia

**Keywords:** alcohol dependence, impulsivity, craving, expectancies, CBT, personalized, RCT

## Abstract

**Background:** Tailored psychological interventions based on individual risk factors are likely to improve treatment for Alcohol Use Disorders (AUDs). Key risk factors for poor treatment outcome include alcohol craving, positive expectations of alcohol consumption, and impulsivity.

**Design:** Pragmatic randomized Cognitive-Behavioral Treatment (CBT) trial.

**Setting:** Public hospital alcohol and drug clinic.

**Participants:** Three-hundred seventy-nine patients (65% male; Age_years_ *M* = 44.32, *SD* = 10.75) seeking treatment for AUD.

**Procedure:** Patients were randomly allocated into treatment as usual (TAU) or targeted treatment. Patients in targeted treatment were allocated one of three treatment modules focusing on craving, positive expectancy, or impulsivity based on assessment results. Treatment included eight, 1 h sessions of CBT over 12 weeks delivered by clinical psychologists.

**Hypotheses:** Targeted treatment was expected to have fewer drinking days and consume less alcohol during the treatment period than TAU. Improvement in targeted mechanisms was predicted to be greatest for patients within matched conditions.

**Results:** Patients attended an average of 4.4 sessions with 93 (25%) completing the whole 12-week treatment episode. The mean proportion of drinking days between sessions was 5% with an average consumption of 64 grams of ethanol. No significant effect of targeted treatment was identified on drinking days or consumption. The craving (*b* = −18.97, 95% CI = −31.44, −6.51) and impulsivity (*b* = −26.65, 95% CI = −42.09, −11.22) modules demonstrated significant reductions in their targeted constructs over treatment, above TAU. Only reduction in craving was associated with reduced drinking days [exp(*b)* = 0.958, *p* = 0.003] and alcohol consumption [exp(*b)* = 0.962, *p* = 0.02]. Significant indirect effects for the targeted craving module through craving reduction were identified for reduction in drinking days (β = −0.72, 95% CI = −1.50, −0.158) and alcohol consumption (β = −0.78, 95% CI = −1.72, −0.11).

**Conclusions:** In the context of a public health service, the effectiveness of individualized treatment targeting risk mechanisms identified during pre-treatment assessment was not confirmed. Some evidence was found for improved treatment response to the implementation of a manualized craving module when pre-treatment craving was high.

## Introduction

Improvements in treatment outcomes for Alcohol Use Disorders (AUDs) have been modest despite substantial research (1). Alcohol is among the leading contributors to the global burden of morbidity and disease, with the majority of this burden attributable to AUDs (2–4). Evidence-based interventions for AUD are available, though relapse rates are high (1, 5, 6). About one in five patients remain abstinent 12 months post-treatment (7). Comprehensive reviews of AUD treatment and rehabilitative services conclude that individual differences are likely to determine differential treatment response (5, 8).

Efforts at personalizing psychological AUD treatments have focused on matching patients to treatments. Two large scale studies have been conducted examining differential treatment effects with *a priori* hypotheses of treatment response based on individual characteristics (9, 10). The largest, Project MATCH, recruited 1,726 patients across nine treatment sites. It tested 20 hypothesis regarding interactions between 10 patient characteristics in three manualized treatments—Cognitive-Behavioral Therapy (CBT), Motivational Enhancement Therapy (MET), and Twelve Step Facilitation (9). More recently, the United Kingdom Alcohol Treatment Trial (UKATT) study recruited 742 patients, across two treatment conditions—MET and social and behavior network therapy (11). Five matching hypothesis were proposed some of which were drawn from *post-hoc* findings of Project MATCH. In both studies, patients were randomly allocated to a condition and *a priori* (characteristics) hypotheses were retrospectively examined on treatment completion. Treatment matching was not found to improve outcomes in either study (9, 12, 13). Authors concluded that “…the intuitively appealing notion that treatment matching can appreciably enhance treatment effectiveness has been severely challenged” (14, p. 1690).

As an alternative to matching patients to different treatments based on individual characteristics, the UKATT Research Team emphasize the scope for prospectively tailoring treatments to patients. Eclectic psychotherapy approaches involving personally tailoring therapy are widely applied in the treatment of mental health disorders (15). Case conceptualization and treatment planning are central to psychological interventions but little progress has been made to standardize and assess individually tailored approaches. This is partly due to an absence of replicable research identifying patient characteristics with prognostic value (16). Furthermore, such mechanisms need to be modifiable to be treatment targets. Litten et al. recommend a framework by which target mechanisms can be derived for personalized interventions. They propose alcohol addiction comprises 3-stages: binge-intoxication, withdrawal–negative affect, and preoccupation–anticipation (“craving”) (17). These stages reflect movement of drinking motivated by impulsivity and positive-reinforcement expectations to drinking for relief. This process maps well onto three prominent constructs within psychosocial conceptualizations of AUD: impulsivity (18, 19) alcohol outcome expectancies (20–22), and craving (23). Identifying individual differences in patient profiles comprising these constructs has potential for informing personalized interventions.

Craving is common across Substance Use Disorders (SUDs) with implications in diagnosis, prognosis, and treatment (23–25). Ninety-nine percent of substance abuse treatment agencies surveyed in the U.S. reported that it is useful to consider craving in treatment planning (26). Craving is a major risk factor for relapse, often comprising physiological discomfort, intrusive substance-related cognitions, and affective distress (27, 28). Each of these experiences are recognized targets of cognitive and behavioral interventions (29–31). There is a high level of variance in craving severity among patients presenting for treatment of AUD (25), making it a prime candidate for individualized treatment approaches.

Expectations of the outcome of alcohol consumption are central to cognitive and behavioral theories of addiction (20, 21, 32). Positively biased alcohol outcome-expectancies (AOEs) are predictive of drinking initiation, progression to problematic use, and maintenance of AUDs (20, 21). Reduction of positive AOEs is proposed as an important aspect of successful treatment (22, 29, 32, 33) and is commonly targeted within AUD interventions (29, 34, 35). Reduction in positive AOEs is proposed to reduce motivation to drink and enhance drinking refusal self-efficacy (22, 32, 33).

Impulsivity is broadly considered a predisposition for action with insufficient forethought and impaired behavioral restraint (36). Impulsivity is causally linked to heavier alcohol use (37) and predictive of the development of alcohol misuse (38, 39). It is related to poorer SUD treatment outcomes (18, 40–42) and associated with enhanced relapse risk 12-months post-treatment (43, 44). As impulsivity is widely considered a stable trait, the goal of intervention is to improve management and expression of impulsivity (45). Limited research has examined the effectiveness of targeting impulsivity within AUD interventions, although it is recognized as an important avenue for investigation (18, 42, 46).

Identification of individual differences in modifiable mechanisms contributing to the maintenance of AUDs may facilitate individually tailored psychological interventions. Such interventions are expected to improve treatment efficiency and outcome. This study randomly allocated patients seeking treatment for AUD into treatment as usual (TAU) or targeted treatment in a public hospital clinic. Patients in the targeted treatment group were allocated one of three treatment modules—craving, positive expectancy, or impulsivity–depending on pre-treatment assessment results. The targeted treatment condition was expected to have significantly greater retention, fewer lapses during treatment, and less severe lapses than treatment as usual (TAU). Improvements in the mechanisms of interest (craving, AOEs, and impulsivity) were predicted to be greatest for patients within the individually targeted condition.

## Method

The study was a single blind pragmatic randomized control trial with a CBT intervention. Consecutive alcohol dependent patients (subsequently referred to as AUD, consistent with DSM-5 nomenclature) attending a metropolitan hospital outpatient drug and alcohol service were randomly allocated to TAU or targeted treatment. Treatment was administered by registered clinical psychologists (masters level or above). Human research ethics approval was obtained and the trial was registered at the Australian New Zealand Clinical Trials Registry (Trial ID: ACTRN12613000865718).

### Participants

Participants were 379 patients treated consecutively from January 2014 to January 2017. Sample characteristics are presented in Table 1. Inclusion in the trial required that patients be at least 18 years old, meet DSM IV-TR criteria for alcohol dependence and be seeking abstinence as the primary treatment goal (not controlled drinking). Patients were excluded from the study if they had a comorbid substance dependence (with the exception of nicotine), were taking Disulfiram or any prescribed opioid, or if they could not provide written, informed consent.

**Table 1:** Descriptive statistics of the sample at pre-treatment assessment (*n* = 379)^a^.

			**Targeted treatment module**			
	**TAU**	**Targeted treatment**	**Impulsivity**	**Expectancy**	**Craving**	**Total**
	***n* = 193**	***n* = 186**	***n* = 45**	***n* = 64**	***n* = 77**	***n* = 379**
**Mean (SD)**						
**ALCOHOL USE MEASURES**						
AUDIT	28.22 (8.92)	27.79 (8.28)	26.80 (8.22)	27.68 (8.29)	28.47 (8.34)	28.01 (8.60)
SADQ	22.06 (13.33)	23.16 (11.99)	23.53 (11.59)	21.66 (11.98)	24.19 (12.26)	22.6 (12.68)
**TARGETED ASSESSMENTS**						
DEQ-positive	75.97 (10.94)	77.61 (10.10)	74.11 (9.85)	83.17 (10.54)	75.04 (7.74)	76.78 (10.55)
ACE-F	43.27 (29.21)	46.36 (28.69)	40.89 (24.22)	27.03 (23.55)	65.01 (23.16)	44.66 (29.01)
DIS	3.94 (3.28)	5.17 (3.56)	8.71 (2.74)	3.55 (3.12)	3.84 (2.93)	4.42 (3.49)
Age, years	45.26 (10.36)	43.34 (11.09)	41.32 (10.54)	44.72 (12.01)	43.38 (10.56)	44.32 (10.75)
Sessions attended	4.34 (2.71)	4.47 (2.70)	4.49 (2.75)	4.73 (2.73)	4.23 (2.67)	4.4 (2.7)
***n*** **(%)**						
Gender, male	128 (66%)	118 (63%)	26 (58%)	39 (61%)	53 (69%)	246 (65%)
Supplementary pharmacotherapy^b^	63 (33%)	65 (35%)	17 (38%)	23 (36%)	25 (32%)	128 (34%)

a *Continuous data presented as Mean (Standard Deviation)*.

b *Taken acamprosate, naltrexone, or both during treatment*.

### Measures

#### The severity of alcohol dependence questionnaire (SADQ)

The SADQ is a 20-item self-report measure assessing physical withdrawal, affective withdrawal, drinking to relieve withdrawal symptoms, alcohol consumption, and rapidity of reinstatement of alcohol dependence (47). Higher scores are indicative of greater alcohol dependence severity. The SADQ has good test-retest reliability and concurrent validity (47). Internal consistency of the SADQ within the current study was good (Cronbach's α = 0.93, 95% CI = 0.92–0.94).

#### The alcohol use disorders identification test (AUDIT)

The AUDIT interview version was administered by experienced nursing staff at patient intake. The AUDIT comprises 10-items assessing recent alcohol use, symptoms of alcohol dependence, and alcohol related problems. Higher scores suggest greater risk of harmful drinking and likelihood of AUD. Good internal consistency, as well as sensitivity and specificity in the detection of AUDs has been demonstrated (48). Internal consistency of the AUDIT was good within the current study (Cronbach's α = 0.87, 95% CI = 0.85–0.89).

#### The alcohol craving experience questionnaire—frequency (ACE-F)

The ACE-F is an 11-item scale assessing desire related cognitions over the previous week. Participants respond via an 11-point visual analog scale with anchors 0 (not at all) and 10 (constantly/extremely). The ACE-F has demonstrated good construct validity, predictive validity, concurrent validity, discriminant validity, internal reliability, and test-retest reliability (25, 49, 50). Internal consistency of the ACE within the current study was excellent (Cronbach's α = 0.95, 95% CI = 0.94–0.96).

#### Drinking expectancy questionnaire (DEQ)

The DEQ is a 43-item self-report measure assessing positive and negative AOEs. Response options range from one “*Strongly Disagree”* to five “*Strongly Agree”* on a 5 point Likert scale. The DEQ comprises six subscales: *Assertion* (10-items) assesses positive beliefs regarding social confidence and assertiveness; *Sexual Enhancement* (5-items) refers to expectations of enhanced feelings of attractiveness and sexual interest; *Cognitive Change* (4-items) assesses beliefs of improved thought generation and clarity; *Tension Reduction* (4-items) evaluates beliefs about the relaxing effects of alcohol; *Affective Change* (12-items) measures beliefs regarding the effects of alcohol on negative mood states, such as sadness, irritability, and aggressiveness; and *Dependence* (8-items) reflects beliefs regarding a personal sense of addiction and perceived loss of control when drinking. The *Assertion, Sexual Enhancement, Cognitive Change, and Tension Reduction* sub-scales are positive AOEs, as they represent positive expectations of alcohol, while the *Affective Change* scale is a negative AOE. As the *Dependence* scale assesses beliefs beyond the acute effects of alcohol, it is considered to be a broader construct than the other scales (51). The DEQ has sound internal consistency and test-re-test reliability, and good construct, convergent, and predictive validity (52). The positive sub-scales of the DEQ were combined to form a proxy for total “positive expectancies.” Combination of all positive expectancy items demonstrated acceptable internal consistency (Cronbach's α = 0.78, 95% CI = 0.75–0.81).

#### Dickman's impulsivity inventory—dysfunctional impulsivity scale (DIS)

The DIS is a sub-scale of Dickman's Impulsivity Inventory, assessing the tendency to act with a lack of forethought when this tendency is a source of difficulty; (53). The DIS comprises 12-items with dichotomous (True/False) response options. The DIS has demonstrated good internal reliability, construct validity, and excellent concurrent validity with other established impulsivity scales (53, 54). The DIS demonstrated good internal consistency within the current study (Cronbach's α = 0.84, 95% CI = 0.81–0.86).

#### Drinking behavior

Patient were asked to recall days of alcohol consumption since the previous treatment session and estimate the quantity consumed on each drinking occasion, guided by the time-line follow-back procedure (55). Drinking days was standardized by dividing the total abstinent days by the number of days the patient was in treatment.

### Procedure

Patients were referred to the alcohol and drug service following inpatient hospital admission, or by their General Practitioner or self-referral. Intake was conducted by a nurse or social worker, who determined eligibility for the AUD treatment program. This included obtaining patient consent and completion of the pre-treatment assessments. No incentive was offered to patients enrolled in the trial. Patients were advised to remain abstinent and scheduled to begin treatment within 7-days. Randomization to treatment condition (TAU or Targeted) was based on a random number sequence generated by program “Research Randomizer” version 4.0 (56). Randomization order was concealed from enrolling and treating staff by a research assistant, who revealed treatment condition upon completion of pre-treatment assessments. Treatment comprised eight face-to-face, one-on-one, sessions of CBT over 12-weeks. Targeted treatment involved administration of one of three manualized modules targeting craving, AOEs, or impulsivity over four of the eight sessions. Module selection was based on highest standardized pre-treatment score on the ACE-F, DEQ, or DIS respectively. Scores were standardized relative to AUD norms to enable comparison across scales. Targeted treatment modules were adapted from existing treatment manuals of evidence-based cognitive-behavioral therapies by registered clinical psychologists with extensive experience treating AUDs. Treating psychologists were trained in module administration. Assessment reports of the targeted treatment scales were provided to psychologists if their patients were allocated to the targeted condition. Psychologists recorded drinking behavior and adjunct pharmacotherapy (naltrexone, acamprosate, or both) at each session. The assessment battery was readministered at 12-weeks. It must be emphasized that this was a pragmatic trial, intended to examine effectiveness of targeted treatment within a “real-world” setting, enhancing external validity (57). Psychologists adhered as closely to the targeted treatment modules as possible, though were free to diverge according to their clinical judgment, such as addressing high risk situations. Psychologists recorded how closely they adhered to the treatment manual (0 = no fidelity to 10 = complete fidelity) for patients enrolled in targeted treatment.

#### Intervention summary

TAU was CBT, where common treatment elements include motivational interviewing, psychoeducation, identification of risks for relapse, problem-solving skills training, relaxation strategies, and relapse prevention planning (29, 58–60). The craving module comprised: craving education, self-monitoring of craving and urges, short-term behavioral strategies for coping with craving, and cognitive strategies for craving management (58, 61). The expectancy module aimed to educate patients about how learned alcohol outcome expectancies contribute toward development and maintenance of drinking problems (32), facilitate development of behavioral alternatives to positive expectancies (e.g., relaxation training, social-skills training), and challenge positively-biased expectancies through cognitive strategies (29). The Impulsivity module was process oriented, intending to help patients recognize the benefits of planning and considered deliberation. This was achieved through the use of clear session structure and documentation to encourage reflection. Specific components of the module included education about impulsivity, cognitive and behavioral response-inhibition strategies (62), structured problem solving (63), and identification of alternative rewards. Module manuals are available upon request.

#### Statistical analysis

Data analysis were conducted in R version 3.3.3. Assessment scores were standardized by percentage of maximum possible score (POMP) to facilitate interpretation and comparison across scales (64). Assumptions of normality and linearity were assessed via inspection of residual and diagnostic plots.

##### Power analysis

A previous study at this treatment site, using an identical generic CBT treatment program (TAU Condition) demonstrated medium effect sizes to detect treatment group differences (65). Accepting an effect size of 0.3 (*w*), *a* = 0.05 and power of 0.90, a minimum of 117 subjects was required for a between group contrast (Critical Chi = 3.84).

##### Treatment effects

The primary outcome of interest was drinking behavior over treatment. Drinking behavior was operationalized as two outcomes, the proportion of drinking days and quantity of alcohol consumed between sessions. Separate analyses were conducted for each drinking behavior. Inspection of the proportion of drinking days and consumption variables revealed zero-inflated heavy tailed positively skewed distributions. Tweedie Compound Poisson models were utilized as they are appropriate for analysis of non-negative continuous data with inflation in discrete zeros (66–68). Data were hierarchically structured, with drinking behavior between sessions nested within patients. Compound Poisson generalized linear mixed models (CPGLMM), treating “patient” as a random effect with “session number” (1–8) nested within, enabled assessment of within and between patient differences in drinking behaviors (intercepts) as well as within and between patient changes in drinking behavior over treatment (slopes). All models were fit using Laplace approximated maximum likelihood estimation in R (version 3.3.3) package “cplm” (66).

All available data were included in the models. Baseline models included a random intercept and “session number.” Covariates: age, gender, severity of alcohol dependence, and medication use (acamprosate, naltrexone, or both) were included sequentially as fixed effects, and retained if they significantly improved model fit as assessed by likelihood ratio tests (69). Pattern-mixture modeling was used to identify and model potential differences in outcomes between patients who dropped out and those who completed treatment by including patient completion (No or Yes) as a fixed effect (70, 71). Moderating effects of each predictor on the trajectory of drinking behavior were assessed by adding the interaction between each predictor and session number to the model. Upon completion of the “covariates model,” pre-treatment targeted assessment scores (DEQ-Positive, ACE-F, DIS) and corresponding assessment by session number interactions were included to control for bias in module allocation pertaining to inflation in these constructs. This formed the “control model.”

Effects of treatment condition were examined by including condition (TAU or Targeted Treatment) as a fixed effect within the control model. Effects of condition on the trajectories of drinking behavior were assessed by including interaction between treatment condition and session number in the model. In a separate model, differential effects among the targeted treatment modules were examined by including a nominal module variable (TAU, Impulsivity, Expectancy, or Craving) to the control model and a session number x module interaction term in the following step. Statistical significance of fixed effects within the final models was estimated by Type III Wald tests in order to partition variance of effects simultaneously comprising part of an interaction.

##### treatment mechanisms

Mediation of the effect of treatment module on drinking behaviors by reduction in the targeted constructs (pre to post-treatment) was tested using the joint significance procedure (72). Support for mediation would be drawn from a significant association between treatment module assignment and reduction in the targeted construct (path *a*), and a significant association between reduction in the targeted construct and drinking behavior (path *b*). Separate mediation models were tested for each treatment module and each drinking behavior, resulting in six mediation analyses.

Path *a* was assessed by separate linear mixed models (LMM) using R package “lme4” (73). Cases without post-treatment targeted assessments were excluded from the analyses (complete cases, *n* = 93). The outcomes were targeted assessment (craving = ACE-F; impulsivity = DIS; positive expectancy = DEQ) nested within assessment occasion (0 = pre-treatment, 1 = post-treatment). “Patient” was modeled as a random effect. Models included a random intercept, assessment occasion (slope), covariates (age, sex, and pre-treatment severity of dependence score), treatment module, and a “treatment module” × “pre-treatment targeted assessment” interaction term. The effect of interest (path *a*) was represented by the interaction between “assessment occasion: post-treatment” and “treatment module.” An additional interaction term comprising pre-treatment ACE-F score and medication-use was included in the craving model to control for potential differences in conjoint pharmacotherapy as a function of pre-treatment craving severity. Models were fit using Maximum Likelihood estimation. Significance was approximated by Wald based 95% confidence intervals around fixed effects. Random slopes were not assessed.

Path *b* was assessed by separate CPGLMMs on the complete cases. Covariate models were constructed using identical procedures to the *Treatment Effects* analyses. All “pre-treatment targeted assessment scores” and “pre-treatment targeted assessment × session number” interaction terms were held constant in all models. “Post-treatment targeted assessment” was included in the final step. As “pre-treatment targeted assessment” scores were held constant within the models, the “post-treatment targeted assessment score” provided a proxy for residualized change in the targeted assessment (path *b*). As the temporal sequence of the predictor in path b (change in target mechanism) is measured after the outcome (drinking behaviors), it is not possible to infer a causal relationship between reduction in target mechanisms and reduction in drinking behavior. In fact, the relationship between mechanism reduction and reduction in drinking is expected to be bidirectional. However, differences between the targeted modules and TAU observed in these pathways will provide insight into the hypothesized mechanisms of action.

Standardized indirect effects were estimated using R package “Rmediation” (74). Confidence intervals were estimated by the product of confidence limits for indirect effects (PRODCLIN) procedure (75). Complete *Treatment Effects* models were constructed on the complete cases to assess consistency of results.

##### Disclosure of divergence to planned analyses

The present analysis method deviates from that proposed in the 2013 trial registration. Advanced statistical methods better suited to modeling this data became accessible through developments in statistical software [e.g., (62)]. In contrast to the planned analyses these developments enabled modeling of patient attrition, distributional properties of the outcomes, covariates, and differences in growth of drinking behavior over treatment. In doing so, error variance was minimized and statistical power maximized (76).

## Results

### Descriptive statistics

**Figure 1:**
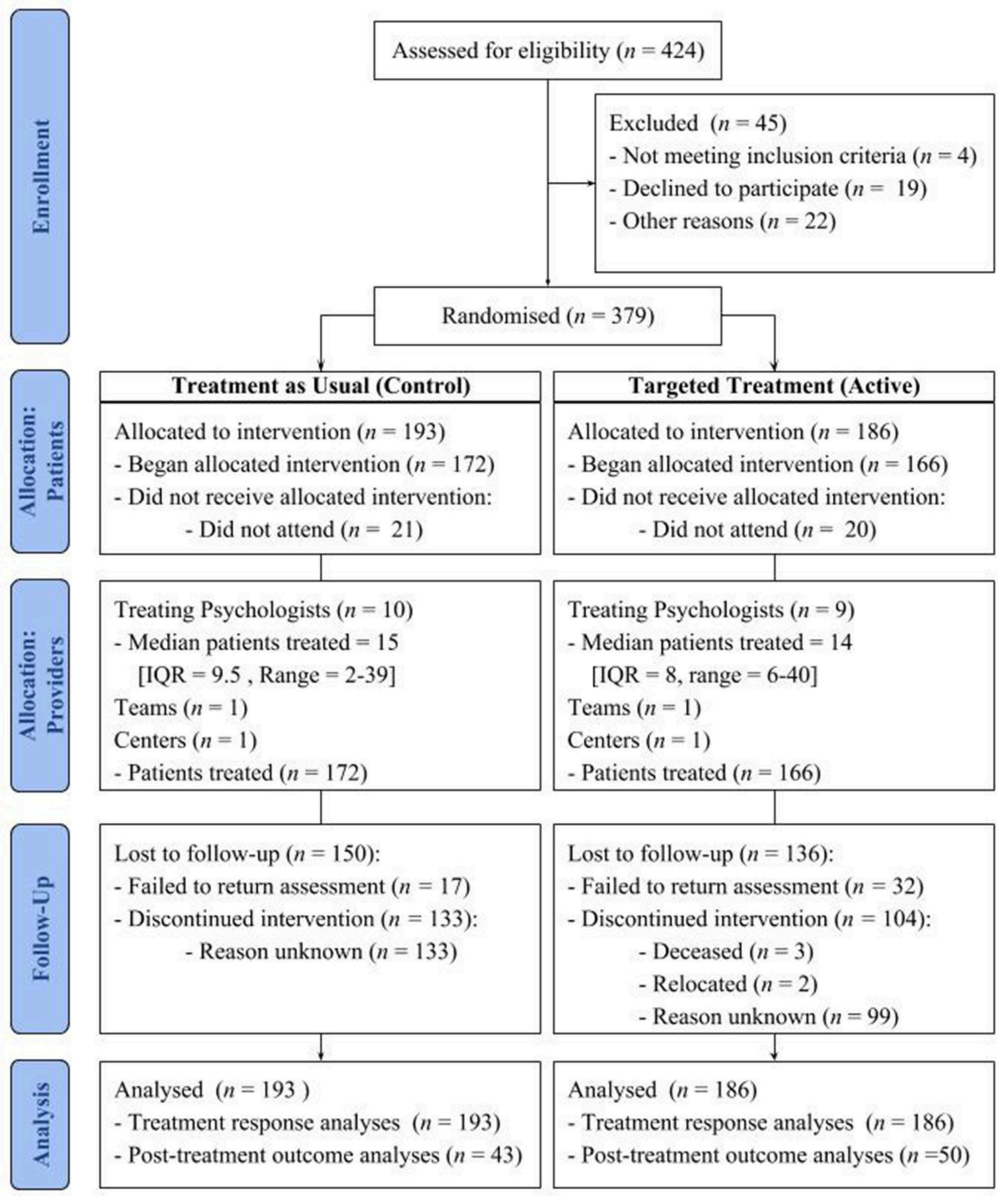
CONSORT flow diagram of patient trial recruitment and retention modified for individual randomized controlled trials of nonpharmacologic treatments (79).

Three-hundred and ninety-seven patients consented to the trial and completed pre-treatment assessments. Ninety-seven percent (*n* = 367) scored = >6 on the AUDIT, indicating a likely AUD (77, 78). Thirty-three percent (*n* = 128) scored < = 15 on the SADQ, indicating mild dependence; 40% (*n* = 153) scored = > 16 and < = 30 indicating moderate dependence; and 26% (*n* = 98) scored = >31, indicating severe dependence. Three-hundred and thirty-eight patients (11%) attended at least one session, with 93 (25%) re-assessed at 3 months. No significant differences in the proportion of patients who completed treatment were observed between TAU and Targeted Treatment groups [$\chi_{\left(1\right)}^{2}$ = 0.4, *p* = 0.53] or treatment modules [Impulsivity $\chi_{\left(1\right)}^{2}$ = 0.57, *p* = 0.45; Expectancy $\chi_{\left(1\right)}^{2}$ = 0.6, *p* = 0.44; Craving $\chi_{\left(1\right)}^{2}$ = 0.004, *p* = 0.95]. The mean sessions attended was 4.4 (*SD* = 2.70). No significant differences in the number of sessions attended were observed between TAU and Targeted Treatment (*U* = 17970.00, *p* = 0.616) or treatment modules [$\chi_{\left(3\right)}^{2}$ = 1.26, *p* = 0.738]. A CONSORT flow chart of patient recruitment and retention is provided in Figure 1, as per Boutron et al. . The mean proportion of drinking days between sessions was 5% (*SD* = 14.71) and mean consumption was 64 (*SD* = 309.41) grams of ethanol. Post-treatment fidelity ratings were available from 50 patients. Mean fidelity rating for the targeted treatment condition was 6.26 (*SD* = 2.39) out of 10. One-way analysis of variance was not indicative of significant differences in fidelity among the treatment modules [*F*_(2, 47)_ = 0.84, *p* = 0.453].

### Part 1: main effects of condition

Separate CPGLMM control models were constructed for the outcomes “proportion of drinking days” and “quantity of consumption.” Superiority of targeted treatment to TAU was assessed by adding treatment condition (TAU, Targeted Treatment) to each control model. Differential effects among targeted modules were similarly examined by adding module (TAU, Impulsivity, Expectancy, Craving) to each control model. Results of these four models are summarized in Table 2.

**Table 2:** Summary of final models assessing the effect of targeted treatment on treatment response^a^^,^^b^.

**Outcome:**	**Proportion of drinking days**						**Consumption**					
**Treatment Predictor:**	**Control (none)**		**Condition**		**Module**		**Control (none)**		**Condition**		**Module**	
	***b (SE)***	***P***	***b* (*SE*)**	***p***	***b* (*SE*)**	***p***	***b (SE)***	***p***	***b* (*SE*)**	***p***	***b* (*SE*)**	***p***
**FIXED EFFECTS**												
Constant	3.03 (3.33)	0.004	2.45 (3.37)	0.002	4.39 (3.58)	0.014	227.65 (5.29)	0.001	184.14 (5.38)	0.002	180.32 (5.93)	0.004
Session	1.64 (1.36)	0.103	1.74 (1.36)	0.07	1.44 (1.39)	0.267	0.98 (1.33)	0.954	1.03 (1.34)	0.928	1.04 (1.38)	0.906
**COVARIATES**												
Sex (male)	0.49 (1.29)	0.005	0.48 (1.29)	0.005	0.46 (1.29)	0.003	0.42 (1.36)	0.005	−0.87 (0.31)	0.005	−0.91 (0.31)	0.003
Sex (Male)*Session	*ns*	*ns*	*ns*	*ns*	*ns*	*ns*	*ns*	*ns*	*ns*	*ns*	*ns*	*ns*
Age	*ns*	*ns*	*ns*	*ns*	*ns*	*ns*	0.97 (1.01)	0.026	0.97 (1.01)	0.028	0.97 (1.01)	0.025
Age*Session	0.99 (1.00)	0.042	0.96 (1.02)	0.044	0.96 (1.02)	0.04	*ns*	*ns*	ns	*ns*	ns	ns
SADQ	*ns*	*ns*	*ns*	*ns*	*ns*	*ns*	*ns*	*ns*	*ns*	*ns*	*ns*	*ns*
SADQ*Session	*ns*	*ns*	*ns*	*ns*	*ns*	*ns*	*ns*	*ns*	*ns*	*ns*	*ns*	*ns*
Pharmacotherapy (Yes)	*ns*	*ns*	*ns*	*ns*	*ns*	*ns*	*ns*	*ns*	*ns*	*ns*	*ns*	*ns*
Pharmacotherapy (Yes)*Session	*ns*	*ns*	*ns*	*ns*	*ns*	*ns*	*ns*	*ns*	*ns*	*ns*	*ns*	*ns*
Complete Tx (Yes)	*ns*	*ns*	*ns*	*ns*	*ns*	*ns*	*ns*	*ns*	*ns*	*ns*	*ns*	*ns*
Complete Tx (No)*Session	*ns*	*ns*	*ns*	*ns*	*ns*	*ns*	*ns*	*ns*	*ns*	*ns*	*ns*	*ns*
**PRE-TX TARGET ASSESSMENTS**												
ACE-F	1.02 (1.01)	0.003	1.02 (1.01)	0.004	1.02 (1.01)	0.017	1.02 (1.01)	0.005	1.02 (1.01)	0.006	1.02 (1.01)	0.03
DIS	1.00 (1.01)	0.927	1.00 (1.01)	0.815	1.00 (1.01)	0.599	1.00 (1.01)	0.612	1.00 (1.01)	0.694	1.00 (1.01)	0.717
DEQ-positive	0.98 (1.02)	0.181	0.98 (1.02)	0.194	0.97 (1.02)	0.095	0.95 (1.02)	0.022	0.95 (1.02)	0.025	0.95 (1.02)	0.054
ACE-F*Session	1.00 (1.00)	0.054	1.00 (1.00)	0.085	1.00 (1.00)	0.016	1.00 (1.00)	0.031	1.00 (1.00)	0.036	1.00 (1.00)	0.03
DIS*Session	1.00 (1.00)	0.029	1.00 (1.00)	0.018	1.00 (1.00)	0.089	1.00 (1.00)	0.101	1.00 (1.00)	0.083	1.00 (1.00)	0.072
DEQ-Positive*Session	1.00 (1.00)	0.748	1.00 (1.00)	0.689	1.00 (1.00)	0.622	1.00 (1.00)	0.234	1.00 (1.00)	0.264	1.00 (1.01)	0.338
**TREATMENT CONDITION**												
Condition (Targeted)			1.57 (1.38)	0.162					1.40 (1.45)	0.364		
Condition (Targeted)*Session			0.90 (1.07)	0.096					0.94 (1.08)	0.422		
**TREATMENT MODULE**												
Module (Impulsivity)					0.81 (1.81)	0.265					1.28 (1.94)	0.800
Module (Expectancy)					2.09 (1.63)						1.23 (1.78)	
Module (Craving)					1.72 (1.53)						1.61 (1.65)	
Module (Impulsivity)*Session					0.93 (1.13)	0.112					0.88 (1.14)	0.728
Module (Expectancy)*Session					0.78 (1.11)						0.93 (1.12)	
Module (Craving)*Session					1.00 (1.09)						1.00 (1.11)	
**RANDOM EFFECTS**												
Patient	46.47 (7.09)		46.16 (7.08)		41.41 (6.89)		165.49 (9.59)		165.33 (9.58)		173.98 (9.69)	
Session	1.03 (1.2)		1.03 (1.19)		1.03 (1.18)		1.03 (1.19)		1.03 (1.19)		1.03 (1.19)	
Residual	1.43 (1.82)		1.43 (1.82)		1.43 (1.82)		2.93e+18679.22)		2.87e+18 (678.25)		2.879e+18 (678.26)	

a *Coefficients presented are exponentiated from the original CPGLMM model and may be interpreted as: each unit increase in the predictor is associated with change in the outcome by the product of 1 × b*.

b *All scale scores are standardized by the percentage of maximum possible score (0–100)*.

*ns indicates that inclusion of the term did not significantly improve model fit (p < 0.05)*.

*Tx, Treatment; SADQ, Severity of Alcohol Dependence Questionnaire; DEQ, Drinking Expectancy Questionnaire; ACE-F, Alcohol Craving Experience - Frequency Scale; DIS, Dysfunctional Impulsivity Scale*.

#### Proportion of drinking days

After controlling for covariates and baseline targeted assessments, neither drinking behavior significantly changed over the course of treatment. Each year increase in age was associated with a 1% reduction in the proportion of drinking days per session attended. Each unit increase in POMP DIS score was associated with a 0.03% increase in proportion of drinking days per session. Being male was associated with a 51% lower proportion of drinking days over treatment. Each unit increase in the ACE-F was associated with 2% increase in total proportion of drinking days. No significant effects of dependence severity, age, pharmacotherapy, or completion status on model intercepts or slopes were identified. No significant effect of treatment condition or treatment module was identified on the intercept or trajectory of the number of drinking days during treatment.

#### Consumption

Progression of sessions attended was not independently associated with an increase in alcohol consumption, but was moderated by POMP ACE-F score with each unit increase associated with a 0.04% reduction in consumption per session attended. Independent of session, each unit increase in POMP ACE-F score was associated with a 2% increase in total alcohol consumption. A unit increase in POMP DEQ-Positive was associated with a 5% overall reduction in consumption over the course of treatment. Being male was associated with 48% less alcohol consumption over treatment (*p* = 0.007). Each year increase in age was associated with a 3% reduction in alcohol consumption during treatment. No significant effects of dependence severity, pharmacotherapy, or completion status on model intercepts or slopes were identified. Treatment condition and module were not significantly related to overall consumption over treatment or progression of consumption over treatment.

### Part 2: mechanisms of change

#### Path a

**Figure 2:**
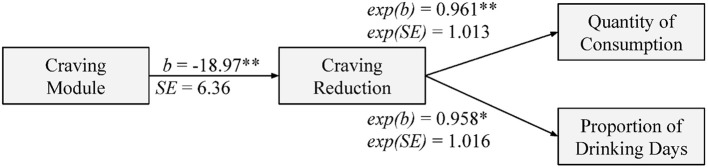
Path figure of the effect of the craving treatment module on drinking behaviors as mediated by change in craving. Craving was standardized as percentage of maximum possible ACE-F score (0–100). Each unit reduction in craving is associated with a reduction in drinking behavior by the product of 1 × exp(*b*). *p < 0.05, **p < 0.01.

LMMs holding covariates and baseline ACE-F score, indicated the craving module was predictive of a 18.97 (*SE* = 6.36, 95% CI = −31.44, −6.51) point reduction in ACE-F score (Figure 2). This effect occurred above a significant main effect of TAU (*b* = −16.52, *SE* = 4.27, 95% CI = −24.89, −8.14). The impulsivity module was predictive of a 26.65 (*SE* = 7.87, 95% CI = −42.09, −11.22) reduction in DIS score post-treatment, above the non-significant effect of TAU (*b* = −4.51, *SE* = 3.75, 95% CI = −11.86, 2.83). The Expectancy treatment module was not significantly predictive of post-treatment Positive DEQ score (*b* = −1.45, *SE* = 2.66, 95% CI = −8.08, 4.02), above the significant effect of TAU (*b* = −7.74, *SE* = 1.47, 95% CI = −10.62, −4.86).

#### Path b

Reconstructed CPGLMM models on the complete cases were consistent with the full dataset, where treatment condition was not significantly predictive of proportion of drinking days or quantity of alcohol consumption over treatment. Adding post-treatment ACE-F score to the covariates models significantly improved fit with both drinking days [exp(*b)* = 1.04, *p* = 0.003] and quantity of alcohol consumption as outcomes [exp(*b)* = 1.04, *p* = 0.020]. Each unit increase in residualized post-treatment POMP ACE-F was associated with a 4% increase in the proportion of drinking days and 3.8% increase in alcohol consumption. Residualized post-treatment ACE-F was not found to affect growth in drinking days [exp(*b)* = 1.00, *p* = 0.353] or alcohol consumption [exp(*b)* = 1.00, *p* = 0.243] over treatment. Post-treatment POMP DIS and DEQ-Positive scores were not significantly related to the intercepts or slopes of either drinking behavior over treatment. As the craving module predicted significant reductions in craving score above TAU (path *a*) and reductions in craving significantly predicted less alcohol consumption and less drinking days over treatment, the joint significance procedure indicates there is evidence for mediation. This was further supported by significant standardized indirect effects of the craving module on alcohol consumption (β = −0.78, *SE* = 0.415, 95% CI = −1.72, −0.11) and drinking days (β = −0.72, *SE* = 0.347, 95% CI = −1.50, −0.158).

## Discussion

The study compared the effectiveness of standard CBT for AUD to a tailored CBT treatment program based on the psychometric profiles of three mechanisms—craving, positive expectancy, and impulsivity–in a public health clinic. Contrary to hypotheses, no significant effect of targeted treatment on patient retention, proportion of drinking days over treatment, or quantity of alcohol consumption over treatment was identified. Nor were differences observed in the trajectory of drinking behaviors over treatment. Indirect support may be drawn for the craving treatment module. Patients within the craving module demonstrated reductions in craving more than twice those within TAU, and reduction in craving was associated with reduced alcohol consumption and fewer drinking days over treatment. This resulted in a significant indirect effect of the craving module, predicting a 0.78 *SD* reduction in alcohol consumption and 0.72 *SD* reduction in drinking days relative to TAU. This provides some evidence for improved treatment response to the implementation of a manualized craving module when pre-treatment craving is high. However, no evidence was found to support the prediction that targeting positive expectancies or impulsivity based on pre-treatment assessments would be superior to TAU in reducing drinking over treatment.

The impulsivity module, and no other module, significantly reduced reported dysfunctional impulsivity among treatment completing patients. This finding should be interpreted carefully, as impulsivity is widely considered an enduring trait (18). We do not interpret this finding as suggesting that the patients are inherently less impulsive, but rather that the impulsivity module improved patients' ability to manage dysfunctional impulses. Surprisingly, this reduction was not significantly related to reduced drinking behavior over treatment. This may indicate that impulsivity may not be as important to effective treatment response as initially thought. An alternative explanation may be that the relative risk of lapse as predicted by the standardized assessments is not equivalent across measures, as is assumed by the module allocation procedure. Future module assignment may be improved by weighting standard scores relative to established effect sizes for the targeted assessments. Follow up is required to identify whether reduction in reported dysfunctional impulsivity is sustained over time and if it improves long-term outcomes.

The expectancy module did not significantly reduce positive expectancies above TAU. This may suggest that the positive expectancy module did not offer sufficient unique influence over the intended construct. It may be that TAU addresses positive expectancies sufficiently as it stands, or that the effect of the present intervention was too small to detect. Furthermore, reduction in positive expectancy was not significantly associated with reduction in drinking behavior over treatment.

Caution is recommended when interpreting any non-hypothesized effects identified among the covariates within the analyses. As the large number of tests make the likelihood of a false-positive finding among the covariates high, any non-hypothesized effects of interest require careful theoretical consideration subsequent replication.

Conducting a pragmatic trial within a public drug and alcohol outpatient hospital facility was ideal for determining intervention effectiveness in a “real world” setting. However, restrictions of public hospital and ethical protocols limit levels of experimental control. Among the primary limitations were the absence of independent fidelity testing and restrictions in assessment. More frequent assessment of the target construct is required to determine the direction of effect between targeted mechanism change and drinking behavior. There were also restrictions in experimental design as treatment of patients within the targeted treatment module was dictated by pre-treatment assessment results not random allocation. This was to minimize the risk of compromising the treatment efficacy of the targeted treatment modules. For example, a patient who scores very high on craving and very low on impulsivity, but is allocated to the impulsivity module, is unlikely to respond well to treatment. This also increases the likelihood of low treatment fidelity by the treating psychologists. Conclusions drawn from this study are also limited to initial treatment response. Long-term follow ups are not compulsory for patients, many opt not to return, and the data that is available is subject to selection bias.

The ability to appropriately tailor psychotherapy to individual patient characteristics has long been recognized as crucial to the progression of treatment efficacy (15). Standardized methods of treatment matching, and in this study, tailoring treatment, have yet to demonstrate utility in the treatment of AUD (13, 80). This study provides evidence for implementing standardized craving modules for patients with high craving pre-treatment. More nuanced understanding of mechanisms of risk, change, and moderators of treatment response are required to enhance standardized approaches to tailoring psychotherapy.

## Ethics statement

This study was carried out in accordance with the recommendations of the National Statement on Ethical Conduct in Human Research, issued by the National Health and Medical Research Council (NHMRC). The protocol was approved by the Metro South Human Research Ethics Committee and The University of Queensland. All subjects gave written informed consent in accordance with the Declaration of Helsinki.

## Author contributions

JPC, MG, RY, and GF were involved in the conceptualization, planning, and implementation of the trial. JPC and MG developed the targeted treatment interventions. JMC was responsible for: management of the trial, including obtaining patient consent and implementing randomization procedures; all data management and statistical analyses; and drafting the manuscript. All authors were involved in the revision of the manuscript.

### Conflict of interest statement

The authors declare that the research was conducted in the absence of any commercial or financial relationships that could be construed as a potential conflict of interest.

## References

[1] Connor JP Haber PS Hall WD. Alcohol use disorders. Lancet (2016) 387:988–98. 10.1016/S0140-6736(15)00122-1 26343838

[2] World Health Organization Global Status Report on Alcohol and Health - 2014 ed. Luxembourg (2014). Available online at: http://www.who.int/substance_abuse/publications/global_alcohol_report/msbgsruprofiles.pdf

[3] Rehm J Dawson D Frick U Gmel G Roerecke M Shield KD. Burden of disease associated with alcohol use disorders in the United States. Alcohol Clin Exp Res. (2014) 38(4):1068–77. 10.1111/acer.12331 24428196 PMC4147870

[4] Rehm J Anderson P Barry J Dimitrov P Elekes Z Feijão F. Prevalence of and potential influencing factors for alcohol dependence in europe. Eur Addict Res. (2015) 21:6–18. 10.1159/000365284 25342593

[5] Miller WR Wilbourne PL. Mesa Grande: a methodological analysis of clinical trials of treatments for alcohol use disorders. Addiction (2002) 97:265–77. 10.1046/j.1360-0443.2002.00019.x 11964100

[6] Assanangkornchai S Srisurapanont M. The treatment of alcohol dependence. Curr Opin Psychiatry. (2007) 20:222–7. 10.1097/YCO.0b013e3280fa837d 17415073

[7] Miller WR Walters ST Bennett ME How effective is alcoholism treatment in the United States? J Stud Alcohol. (2001) 62:211–20. 10.15288/jsa.2001.62.211 11327187

[8] Institute of Medicine Broadening the Base of Treatment for Alcohol Problems [Internet]. Washington, DC: National Academy Press (1990). Available online at: http://content.wkhealth.com/linkback/openurl?sid=WKPTLP:landingpage&an=00005053-199206000-00021

[9] Project MATCH Research Group Matching alcoholism treatments to client heterogeneity: project MATCH posttreatment drinking outcomes. J Stud Alcohol Drugs (1997) 58:7–29. 10.15288/jsa.1997.58.7 8979210

[10] UKATT Research Team United Kingdom Alcohol Treatment Trial (UKATT): hypotheses, design and methods. Alcohol Alcohol. (2001) 36:11–21. 10.1093/alcalc/36.1.11 11139410

[11] UKATT Research Team Effectiveness of treatment for alcohol problems: findings of the randomised UK alcohol treatment trial (UKATT). BMJ (2005) 331:541 10.1136/bmj.331.7516.541 16150764 PMC1200586

[12] Project MATCH Research Group Matching alcoholism treatments to client heterogeneity: treatment main effects and matching effects on drinking during treatment. J Stud Alcohol. (1998) 59:631–9. 10.15288/jsa.1998.59.631 9811084

[13] UKATT Research Team UK Alcohol Treatment Trial: client-treatment matching effects. Addiction (2007) 103:228–38. 10.1111/j.1360-0443.2007.02060.x 18070238

[14] Allen J Anton RF Babor TF Carbonari J Carroll KM Connors GJ Project MATCH secondary a priori hypotheses. Addiction (1997) 92:1671–98. 10.1111/j.1360-0443.1997.tb02889.x 9581001

[15] Norcross JC Wampold BE. What works for whom: tailoring psychotherapy to the person. J Clin Psychol. (2011) 67:127–32. 10.1002/jclp.20764 21108312

[16] Adamson SJ Sellman JD Frampton CM. Patient predictors of alcohol treatment outcome: a systematic review. J Subst Abuse Treat. (2009) 36:75–86. 10.1016/j.jsat.2008.05.007 18657940

[17] Litten RZ Ryan ML Falk DE Reilly M Fertig JB Koob GF. Heterogeneity of alcohol use disorder: Understanding mechanisms to advance personalized treatment. Alcohol Clin Exp Res. (2015) 39:579–84. 10.1111/acer.12669 25833016

[18] Loree AM Lundahl LH Ledgerwood DM. Impulsivity as a predictor of treatment outcome in substance use disorders: review and synthesis. Drug Alcohol Rev. (2015) 119–34. 10.1111/dar.12132 24684591

[19] Dawe S Gullo MJ Loxton NJ. Reward drive and rash impulsiveness as dimensions of impulsivity: Implications for substance misuse. Addict Behav. (2004) 29:1389–405. 10.1016/j.addbeh.2004.06.004 15345272

[20] Jones BT Corbin W Fromme K. A review of expectancy theory and alcohol consumption. Addiction (2001) 96:57–72. 10.1080/09652140020016969 11177520

[21] Monk RL Heim D. A critical systematic review of alcohol-related outcome expectancies. Subst Use Misuse. (2013) 48:539–57. 10.3109/10826084.2013.787097 23647167

[22] Coates JM Gullo MJ Feeney GFX Young RM Dingle GA Connor JP. Alcohol expectancies pre-and post-alcohol use disorder treatment: clinical implications. Addict Behav. (2018) 80:142–9. 10.1016/j.addbeh.2018.01.029 29407685

[23] Tiffany ST Wray JM. The clinical significance of drug craving. Ann NY Acad Sci. (2012) 1248:1–17. 10.1111/j.1749-6632.2011.06298.x 22172057 PMC4041083

[24] American Psychiatric Association Diagnostic and Statistical Manual of Mental Disorders. 5th ed Washington, DC: American Psychiatric Association (2013).

[25] Coates JM Gullo MJ Feeney GFX Kavanagh DJ Young RM Dingle GA. The mini alcohol craving experience questionnaire: development and clinical application. Alcohol Clin Exp Res. (2017) 41:156–64. 10.1111/acer.13278 28019645

[26] Pavlick M Hoffmann E Rosenberg H A nationwide survey of American alcohol and drug craving assessment and treatment practices. Addict Res Theory (2009) 17:591–600. 10.3109/16066350802262630

[27] Bottlender M Soyka M. Impact of craving on alcohol relapse during, and 12 months following, outpatient treatment. Alcohol Alcohol. (2004) 39:357–61. 10.1093/alcalc/agh073 15208171

[28] Kavanagh DJ Andrade J May J. Imaginary relish and exquisite torture: The elaborated intrusion theory of desire. Psychol Rev. (2005) 112:446–67. 10.1037/0033-295X.112.2.446 15783293

[29] Beck AT Wright FD Newman CF Liese BS Cognitive Therapy of Substance Abuse. New York, NY: Guilford Press (1993). 8289917

[30] Marlatt GA Gordon JR Relapse Prevention: Maintenance Strategies in the Treatment of Addictive Behaviors. New York, NY: Guilford Press (1985). Available online at: http://www.loc.gov/catdir/toc/ecip056/2005000834.html

[31] Witkiewitz K Bowen S Douglas H Hsu SH. Mindfulness-based relapse prevention for substance craving. Addict Behav. (2013) 38:1563–71. 10.1016/j.addbeh.2012.04.001 22534451 PMC3408809

[32] Bandura A A sociocognitive analysis of substance abuse: An agentic perspective. Psychol Sci. (1999) 10:214–7. 10.1111/1467-9280.00138

[33] Gullo MJ Dawe S Kambouropoulos N Staiger PK Jackson CJ. Alcohol expectancies and drinking refusal self-efficacy mediate the association of impulsivity with alcohol misuse. Alcohol Clin Exp Res. (2010) 34:1386–99. 10.1111/j.1530-0277.2010.01222.x 20528818

[34] Ellis A Reason and Emotion in Psychotherapy. Commentary. New York, NY: Lyle Stuart inc (1962).

[35] Meichenbaum D Cognitive-Behavior Modification. New York, NY: Plenum Press (1977).

[36] Evenden JL. Varieties of impulsivity. Psychopharmacology (1999) 146:348–61. 10.1007/PL00005481 10550486

[37] Gullo MJ Loxton NJ Price T Voisey J Young RMD Connor JP. A laboratory model of impulsivity and alcohol use in late adolescence. Behav Res Ther. (2017) 97:52–63. 10.1016/j.brat.2017.07.003 28715663

[38] Moffitt TE Arseneault L Belsky D Dickson N Hancox RJ Harrington H. A gradient of childhood self-control predicts health, wealth, and public safety. Proc Natl Acad Sci USA. (2011) 108:2693–8. 10.1073/pnas.1010076108 21262822 PMC3041102

[39] George SM Connor JP Gullo MJ Young RM A prospective study of personality features predictive of early adolescent alcohol misuse. Pers Individ Dif. (2010) 49:204–9. 10.1016/j.paid.2010.03.036

[40] Charney D Zikos E Gill KJ. Early recovery from alcohol dependence: factors that promote or impede abstinence. J Subst Abuse Treat. (2010) 38:42–50. 10.1016/j.jsat.2009.06.002 19632079

[41] Rupp CI Beck JK Heinz A Kemmler G Manz S Tempel K. Impulsivity and alcohol dependence treatment completion: is there a neurocognitive risk factor at treatment entry? Alcohol Clin Exp Res. (2016) 40:152–60. 10.1111/acer.12924 26683585

[42] Hershberger AR Um M Cyders M. The relationship between the UPPS-P impulsive personality traits and substance use psychotherapy outcomes: a meta-analysis. Drug Alcohol Depend. (2017) 178:408–16. 10.1016/j.drugalcdep.2017.05.032 28709080 PMC5561735

[43] Evren C Durkaya M Evren B Dalbudak E Cetin R. Relationship of relapse with impulsivity, novelty seeking and craving in male alcohol-dependent inpatients. Drug Alcohol Rev. (2012) 31:81–90. 10.1111/j.1465-3362.2011.00303.x 21450046

[44] Müller SE Weijers HG Böning J Wiesbeck G. Personality traits predict treatment outcome in alcohol-dependent patients. Neuropsychobiology (2008) 57:159–64. 10.1159/000147469 18654085

[45] Butz M Austin S Management of the adult impulsive client: identification, timing, and methods of treatment. In: McCown WG Johnson JL Shure MB editors. The Impulsive Client: Theory, Research, and Treatment. Washington, DC: American Psychiatric Association (1993). p. 323–44.

[46] Czapla M Simon JJ Richter B Kluge M Friederich HC Herpertz S. The impact of cognitive impairment and impulsivity on relapse of alcohol-dependent patients: implications for psychotherapeutic treatment. Addict Biol. (2016) 21:873–84. 10.1111/adb.12229 25678237

[47] Stockwell T Murphy D Hodgson R. The severity of alcohol dependence questionnaire: its use, reliability and validity. Br J Addict. (1983) 78:145–55. 10.1111/j.1360-0443.1983.tb05502.x 6135435

[48] Saunders JB Aasland OG Babor TF de la Fuente JR Grant M. Development of the alcohol use disorders identification test (AUDIT): WHO collaborative project on early detection of persons with harmful alcohol consumption–II. Addiction. (1993) 88:791–804. 10.1111/j.1360-0443.1993.tb02093.x 8329970

[49] Statham DJ Connor JP Kavanagh DJ Feeney GFX Young RMD May J. Measuring alcohol craving: development of the alcohol craving experience questionnaire. Addiction (2011) 106:1230–8. 10.1111/j.1360-0443.2011.03442.x 21438940

[50] May J Andrade J Kavanagh DJ Feeney GFX Gullo MJ Statham DJ. The Craving Experience Questionnaire: a brief, theory-based measure of consummatory desire and craving. Addiction (2014) 109:728–35. 10.1111/add.12472 24400950

[51] Young RM Connor JP Feeney GFX. Alcohol expectancy changes over a 12-week cognitive-behavioral therapy program are predictive of treatment success. J Subst Abuse Treat. (2011) 40:18–25. 10.1016/j.jsat.2010.08.003 20864294

[52] Young RM Oei TPS Manual for the Drinking Expectancy Profile: Incorporating the Drinking Expectancy Questionnaire (DEQ) and the Drinking Refusal Self-Efficacy Questionnaire (DRSEQ). Brisbane, QLD: Behaviour Research and Therapy Centre, University of Queensland (1996).

[53] Dickman SJ. Functional and dysfunctional impulsivity: personality and cognitive correlates. J Pers Soc Psychol. (1990) 58:95–102. 10.1037/0022-3514.58.1.95 2308076

[54] Claes L Vertommen H Braspenning N Psychometric properties of the dickman impulsivity inventory. Pers Individ Dif. (2000) 29:27–35. 10.1016/S0191-8869(99)00172-5

[55] Sobell LC Sobell MB Timeline follow-back. In: Litten RZ Allen J editors. Measuring Alcohol Consumption. Totowa, NJ: Humana Press (1992). p. 41–72. Available online at: http://link.springer.com/10.1007/978-1-4612-0357-5_3

[56] Urbaniak GC Plous S Research Randomizer (Version 4.0) [Computer Software]. (2013). Available online at: https://www.randomizer.org

[57] Thorpe KE Zwarenstein M Oxman AD Treweek S Furberg CD Altman DG. A pragmatic-explanatory continuum indicator summary (PRECIS): a tool to help trial designers. J Clin Epidemiol. (2009) 62:464–75. 10.1016/j.jclinepi.2008.12.011 19348971

[58] Marlatt GA Witkiewitz K Relapse prevention for alcohol and drug problems. In: Marlatt GA Donovan D editors. Relapse Prevention for Alcohol and Drug Problems. New York, NY: Guilford Press (2005). p. 1–44.

[59] Monti PM Abrams DB Kadden RM Cooney NL Treating Alcohol Dependence: A Coping Skills Training Guide. [Internet]. Treating Alcohol Dependence: A Coping Skills Training Guide. New York, NY: Guilford Press (1989). p. 1989 Available online at: http://ovidsp.ovid.com/ovidweb.cgi?T=JS&PAGE=reference&D=psyc3&NEWS=N&AN=1989-98240-000

[60] Miller WR Rollnick S Motivational Interviewing: Helping People Change. 3rd ed New York, NY: Guilford Press (2012).

[61] Kadden R Carroll KM Donovan D Cooney N Monti PM Abrams D Cognitive - Behavioral Coping Skills Therapy Manual. Rockville, MD: National Institute on Alcohol Abuse and Alcoholism Project MATCH Monograph Series (2003).

[62] Linehan MM Skills Training Manual for Treating Borderline Personality Disorder. New York, NY: Guilford Press (1993). Available online at: http://www.loc.gov/catdir/bios/guilford051/93015216.html

[63] D'Zurilla TJ Nezu AM Problem-Solving Therapy. In: Dobson K, editor. Handbook of Cognitive-Behavioral Therapies. 3rd ed New York, NY: Guilford Press (2010). p. 197–225.

[64] Cohen P Cohen J Aiken LS West SG The problem of units and the circumstance for POMP. Multivariate Behav Res. (1999) 34:315–46. 10.1207/S15327906MBR3403_2

[65] Feeney GFX Young RMD Connor JP Tucker J McPherson A. Cognitive behavioural therapy combined with the relapse-prevention medication acamprosate: are short-term treatment outcomes for alcohol dependence improved? Aust N Z J Psychiatry (2002) 36:622–8. 10.1046/j.1440-1614.2002.01019.x 12225445

[66] Zhang Y Likelihood-based and Bayesian methods for Tweedie compound Poisson linear mixed models. Stat Comput. (2013) 23:743–57. 10.1007/s11222-012-9343-7

[67] Dunn PK Smyth GK Series evaluation of Tweedie exponential dispersion model densities. Stat Comput. (2005) 15:267–80. 10.1007/s11222-005-4070-y

[68] Gilchrist R Drinkwater D The use of the Tweedie distribution in statistical modelling. In: Jelke G van der Heijden PGM, editors. Proceedings in Computational Statistics 2000. Heidelberg: Physica-Verlag (2000). p. 313–8.

[69] Royall R Statistical Evidence: A Likelihood Paradigm. New York, NY: Chapman & Hall (1997).

[70] Hedeker D Gibbons RD Application of random-effects pattern-mixture models for missing data in longitudinal studies. Psychol Methods (1997) 2:64–78. 10.1037/1082-989X.2.1.64

[71] Little RJA Modeling the drop-out mechanism in repeated-measures studies. J Am Stat Assoc. (1995) 90:1112.

[72] MacKinnon DP Lockwood CM Hoffman JM West SG Sheets V. A comparison of methods to test mediation and other intervening variable effects. Psychol Methods (2002) 7:83–104. 10.1037/1082-989X.7.1.83 11928892 PMC2819363

[73] Bates D Mächler M Bolker BM Walker SC Fitting linear mixed-effects models using lme4. J Stat Softw. (2015) 67:1–48. 10.18637/jss.v067.i01

[74] Tofighi D MacKinnon DP. RMediation: an R package for mediation analysis confidence intervals. Behav Res Methods (2011) 43:692–700. 10.3758/s13428-011-0076-x 21487904 PMC3233842

[75] MacKinnon DP Fritz MS Williams J Lockwood CM. Distribution of the product confidence limits for the indirect effect: program PRODCLIN. Behav Res Methods (2007) 39:384–9. 10.3758/BF03193007 17958149 PMC2819369

[76] Hallgren KA Witkiewitz K. Missing data in alcohol clinical trials : a comparison of methods. Alcohol Clin Exp Res. (2013) 37:2152–60. 10.1111/acer.12205 23889334 PMC4113114

[77] Lundin A Hallgren M Balliu N Forsell Y. The use of alcohol use disorders identification test (AUDIT) in detecting alcohol use disorder and risk drinking in the general population: validation of AUDIT using schedules for clinical assessment in neuropsychiatry. Alcohol Clin Exp Res. (2015) 39:158–65. 10.1111/acer.12593 25623414

[78] Reinert DF Allen JP. The alcohol use disorders identification test: an update of research findings. Alcohol Clin Exp Res. (2007) 31:185–99. 10.1111/j.1530-0277.2006.00295.x 17250609

[79] Boutron I Altman DG Moher D Schulz KF Ravaud P. CONSORT Statement for randomized Trials of nonpharmacologic treatments: A 2017 update and a CONSORT extension for nonpharmacologic Trial Abstracts. Ann Intern Med. (2017) 167:40–7. 10.7326/M17-0046 28630973

[80] Project MATCH Research Group Matching patients with alcohol disorders to treatments: Clinical implications from Project MATCH. J Ment Heal. (1998) 7:589–602. 10.1080/09638239817743

